# Sini-San Regulates the NO-cGMP-PKG Pathway in the Spinal Dorsal Horn in a Modified Rat Model of Functional Dyspepsia

**DOI:** 10.1155/2020/3575231

**Published:** 2020-03-31

**Authors:** Zhenyu Wu, Xiaofang Lu, Shengsheng Zhang, Chunyang Zhu

**Affiliations:** Digestive Disease Center, Beijing Hospital of Traditional Chinese Medicine, Capital Medical University, Beijing 100010, China

## Abstract

The present study investigated the effect of Chinese medicine Sini-San (SNS) on visceral hypersensitivity in a rat model of functional dyspepsia (FD), and it explored related underlying mechanisms. The rat model of FD was developed by combining neonatal iodoacetamide (IA) treatment and adult tail-clamping. After SNS treatment, the behavior and electromyographic testing were performed to evaluate the visceromotor responses of rats to gastric distention. Immunofluorescence was used to detect the distribution of iNOS-positive cells in the spinal dorsal horn, while the real-time quantitative PCR and western blot were used for detection of the gene expression of c-fos, iNOS, and GABAb and protein levels of iNOS and GABAb in the spinal dorsal horn, respectively. The protein concentration of cGMP and PKG proteins in the spinal dorsal horn were quantified by enzyme-linked immunosorbent assay. In this study, SNS treatment significantly reduced the behavioral score and electromyographic response to graded intragastric distension pressure. The middle-dose of SNS treatment significantly reduced the distribution of iNOS-positive cells in the spinal dorsal horn of FD model rats. The gene expression of c-fos, iNOS, and GABAb and the protein contents of iNOS, GABAb, cGMP, and PKG in the spinal dorsal horn of FD model rats were restored to a normal level by middle-dose of SNS treatment. Our results suggest that Sini-San may alleviate the visceral hypersensitivity in FD model rats via regulation of the NO/cGMP/PKG pathway in the spinal dorsal horn.

## 1. Introduction

Functional dyspepsia (FD) is a chronic disorder of the upper digestive tract characterized by postprandial fullness, early satiation, epigastric pain, and epigastric burning in the absence of organic disease [1]. Existing studies have shown that the prevalence of FD ranges from 9.8% to 40% in Western populations and 5.3%–28% in Eastern populations [2].

The etiology of FD is multifactorial, and the visceral hypersensitivity is one of the major pathophysiologic disturbances [1]. Under the pathological conditions, spinal cord dorsal horn neurons undergo marked plastic changes, eventually leading to hyperactivity of the projection neurons, thus playing an essential role in visceral hypersensitivity and pain [3, 4]. Although many signaling pathways in the spinal dorsal horn, such as the NO/cGMP/PKG pathway, have been confirmed to be related to hyperalgesia [5–7], the treatment for visceral hypersensitivity and FD is still limited and unsatisfactory due to the lack of specific drugs.

Traditional Chinese Medicine (TCM) is an effective alternative treatment for FD [8–16]. According to TCM, FD is divided into different syndromes based on the clinical symptoms and signs, among which “spleen-deficiency and qi-stagnation” is the most common one [17]. In this syndrome, spleen-deficiency is “Ben” (primary aspect), a long-term pathological state related to inappropriate early diet and other adverse early-life experiences. Qi-stagnation is “Biao” (secondary aspect), which is the inducement of worsening symptoms, mostly related to short-term stress. For FD with spleen-deficiency and qi-stagnation syndrome, invigorating spleen and regulating qi are the most appropriate treatment methods, which have shown to produce better treatment results compared with conventional pharmacotherapy [18].

Sini-San (SNS), a representative prescription for invigorating spleen and regulating qi, is commonly used in the treatment of spleen-deficiency and qi-stagnation syndrome in TCM. It contains four herbs, including Chaihu (Radix Bupleuri Chinensis), Baishao (Radix Paeoniae Alba), Zhishi (Fructus Aurantii Immaturus), and Gancao (Radix Glycyrrhizae). Our previous study showed that SNS has certain therapeutic effect in FD rats [19]; however, in that study, the rat model was established by short-term tail-clamping stress, which is not enough to induce chronic FD and “spleen-deficiency and qi-stagnation” syndrome. In order to better simulate the clinical practice, a modified rat model of FD with “spleen-deficiency and qi-stagnation” syndrome was developed by combining neonatal iodoacetamide (IA) treatment and the adult tail-clamping approach [20].

In this study, we used the modified FD rat model to investigate the effect and molecular mechanism of SNS in FD therapy in the spinal dorsal horn.

## 2. Materials and Methods

### 2.1. SNS Preparation

Sini-San (SNS) was prepared as previously described [19]. Briefly, 400 g herbs of Chaihu (voucher number C20181205-01), Baishao (voucher number C20181112-18), Zhishi (voucher number C20180920-01), and Gancao (voucher number C20181220-05) were mixed using a ratio of 1 : 1 : 1 : 1 and impregnated in 2400 ml distilled water for 30 min. Then, 400 ml of liquid medicine was obtained after boiling for 30 minutes. The procedure was then repeated, and another 400 ml of liquid medicine was obtained. A total volume of 800 ml SNS (made from 400 g herbs) was obtained by fully mixing the two 400 ml liquid medicines. Finally, the SNS (400 g herbs/800 ml) was prepared into three concentrations by adding water: low-dose SNS (0.125 g herbs/ml), middle-dose SNS (0.25 g herbs/ml), and high-dose SNS (0.5 g herbs/ml). All herbs were purchased from Beijing Xinglin Pharmaceutical Company and were identified as eligible medicinal material. SNS was prepared by the Beijing Hospital of Traditional Chinese Medicine, Capital Medical University.

### 2.2. Animal Model

The animal protocol was designed to minimize pain or discomfort to the animals. Thirty specific pathogen-free (SPF) male Sprague Dawley (SD) rats (7 day old) were purchased from Beijing Vital River Laboratory Animal Technology Company. All animals used in this study were housed in an SPF animal facility at the China Academy of Chinese Medicine Science and maintained under a 12 h light/12 h dark cycle. All of the rats had free access to food and water and were cared for in accordance with the principles of laboratory animal care approved in China. All animal studies (including the rat euthanasia procedure) were performed in compliance with the regulations and guidelines of the Institute of Basic Theory (China Academy of Chinese Medical Sciences) institutional animal care and conducted according to the AAALAC and the IACUC guidelines (no. 2018-043).

**Figure 1:**
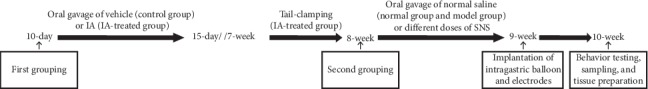
Experimental procedures and time line: 10-day-old rats were treated with 0.1% IA for 6 days. Rats were then normally housed to 7-week-old and performed with tail-clamping for 7 days. After that, 8-week-old rats received normal saline or SNS treatments for 7 days. Then, rats were housed to 9-week-old and received implantation of intragastric balloon and electrodes. At the age of 10 weeks, all rats were performed with behavioral testing before sampling and tissue preparation.

As shown in Figure 1, after 3-day acclimation, the 10-day-old SD rats were randomly divided into 2 groups, including a control group (*n* = 6) and the IA-treated group (*n* = 24). The control group received 0.2 mL of 2% sucrose solution via oral gavage on a daily basis for 6 days, after which they were normally housed until reaching the age of 8 weeks. The IA-treated group received 0.2 mL of 2% sucrose solution with 0.1% IA (1001645681, Sigma-Aldrich) via oral gavage on a daily basis for 6 days, after which they were normally housed until reaching the age of 7 weeks. The 7-week-old IA-treated rats were used to establish the FD model by the tail-clamping approach as previously described [19]. In brief, a long sponge holding forceps was used to clamp the distal one-third of the tail causing no damage to the skin. This tail-clamping was performed every 3 h with a duration of 30 min, 4 times per day for a total of 7 days.

The FD model rats were then randomly allocated into four groups (*n* = 6/group): a model group, which received 1 ml/100 g body weight of normal saline per day; low-dose SNS-treated (low-dose SNS) group that received 1 ml/100 g body weight of low-dose SNS per day; middle-dose SNS-treated (middle-dose SNS) group that received 1 ml/100 g body weight of middle-dose SNS per day, which was equivalently converted from clinical human dose; and high-dose SNS-treated (high-dose SNS) group, which received 1 ml/100 g body weight of high-dose SNS per day. All rats were administered for 7 consecutive days using gavage.

### 2.3. Implantation of Intragastric Balloon and Electrodes

The day after the last gavage, implantation of intragastric balloon and electrodes were performed as previously described [21]. Briefly, after 20-h fasting, 9-week-old rats were weighted and the spherical balloons were implanted in the stomach through an incision at the tip of the fundus under sodium pentobarbital anesthesia (intraperitoneal injection of 100 mg/kg of body weight). The balloon with a maximal volume of 20 ml can be inflated with polyethylene tubing, which was exteriorized at the back of the neck. A pair of stainless steel wires were implanted into the acromiotrapezius muscle and externalized at the back of the neck. The surgery was performed under strict sterile conditions, and the wounds were cleaned with cotton swabs soaked in 75% alcohol. The rats underwent a 2-h water deprivation and 24-h fasting after surgery, after which they were normally fed for a week.

### 2.4. Behavior and Electromyographic Testing

All rats were treated with graded gastric balloon distention, and behavioral responses were evaluated in each one at the age of 10 weeks. The behavioral and electromyographic(EMG) testing was performed as previously described [21]. Briefly, after 20-h fasting, the tested rat was placed in an individual rat restrainer in a quiet environment and allowed to adapt for 40 min. The catheter from the gastric balloon was connected to a barostat, and the steel wires at the back of the rat's neck were connected to a biological and functional experimental system. Then, the rat received graded intragastric pressure (20, 40, 60, 80 mm Hg) by inflating the balloon for 30 s with an interval of 3 min between distensions. The myoelectricity was recorded by the PowerLab/4SP.

Responses of rats to gastric distention were evaluated by simultaneously quantifying EMG activity and behavioral response. EMG activity was determined by the rate of change of root mean square (RMS) value of the EMG signal in the acromiotrapezius. The rate of change of RMS value was calculated using the following formula: (RMS value in the distension period—RMS value in the rest period)/RMS value in the rest period × 100%. Behavioral responses were graded according to the abdominal withdrawal reflex (AWR) as previously described, [21] where 0 stood for no behavioral response to gastric balloon distention; 1 for immobility and brief head movement; 2 for gentle contraction of abdominal muscles without lifting the abdomen; 3 for strong contraction of abdominal muscles and lifting of the abdomen; and 4 for hard contraction of abdominal muscles, body arching, lifting of the abdomen, pelvic structures, and perineum, and stretching of the body. A blinded observer evaluated the AWR score.

### 2.5. Weight and 3-Hour Food Intake

The weight of rats was detected during IA treatment, tail-clamping, and SNS treatment. At the age of 8 weeks, 9 weeks, and 10 weeks, all rats were tested for 3-h food intake. Rats were fasted for 12 h before the test. Each rat was given 30 g of food for 3 h on the next day. The remaining food was recorded after 3 h. According to the formula, the 3-h food intake was calculated using the following formula: *3-h food intake (g)* = *30 (g)*—*the remaining food (g)*.

### 2.6. Immunohistofluorescence Analysis

Twenty minutes after the behavioral and electromyographic testing, rats were deeply anesthetized with sodium pentobarbital (100 mg/kg intraperitoneal). The T8-T10 spinal cord was removed. The spinal cord was postfixed and paraffin was embedded and cut into serial transverse sections (4 *μ*m). Spinal tissue sections were washed with PBS and incubated overnight at 4°C with a primary antibody specific for iNOS (Rabbit Anti-iNOS antibody, 1 : 300, ab178945, Abcam plc.). The primary antibody was detected by incubating the tissue in the goat anti-rabbit antibody (PV-6001, Beijing Zhongshan Jinqiao Biotechnology Co., Ltd) for 40 min. Tissue sections were then mounted on slides and visualized with a fluorescence microscope (OLYMPUS DP71, Japan). The iNOS immunoreactivity was quantified using Image-Pro Plus6.0 software. Briefly, the images were converted to grayscale and black-white reversing processing pattern with Image-Pro Plus6.0. Then, we analyzed the mean value of integrated optical density (IOD) of the images to reflect the intensity of immunostaining.

### 2.7. RNA Isolation and Complementary DNA Synthesis

Twenty minutes after the behavioral and electromyographic testing, rats were deeply anesthetized with sodium pentobarbital (100 mg/kg intraperitoneal). The T8-T10 dorsal spinal cord was removed. The total RNA was extracted from the T8 to T10 dorsal spinal cord of rats using the TRIZOL reagent (Tiangen). The possible genome DNA contamination was first eliminated using the gDNA Eraser contained in the PrimeScript RT reagent kit with a gDNA eraser (Takara), after which cDNA was generated by RT primer mix according to the manufacturer's manual. Briefly, the following conditions were performed in a total volume of 10 *μ*l: 2.0 *μ*l 5 × gDNA Eraser buffer, and 1.0 *μ*l gDNA Eraser was mixed with 1 *μ*g total RNA and incubated 2 min at 42°C to eliminate the genome DNA contamination. Then, 10 *μ*l reverse-transcription mix containing 1.0 *μ*l PrimeScript RT enzyme mix I, 1.0 *μ*l RT Primer Mix containing oligo dT primer and random 6 mers, 5 × PrimeScript buffer 2 was added to the tube and incubated for 15 min at 37°C followed by for 5 sec at 85°C. The product of the cDNA synthesis reaction was stored at −20°C or used for real-time PCR immediately.

### 2.8. Real-Time Quantitative PCR

Gene-specific primers (Table 1) were designed for each tested gene. The real-time quantitative PCR was performed in triplicate using the ABI 7500 real-time PCR instrument (Applied Biosystems) with the TB Green Premix Ex Taq II (Tli RNase H Plus) kit. The PCR experiments were performed according to the protocol and cycling conditions outlined in the manual. Negative control was performed by using cDNA generated without reverse transcriptase as templates. Reactions containing primer pairs without a template were also included as blank controls. The GAPDH gene was used as an internal control to normalize all the other genes. The 2^−ΔΔCt^ method was used to analyze the relative changes in gene expression.

### 2.9. Western Blot Analysis

Twenty minutes after the behavioral and electromyographic testing, rats were deeply anesthetized with sodium pentobarbital (100 mg/kg intraperitoneal). The T8-T10 dorsal spinal cord was removed. The T8 to T10 dorsal spinal cord was homogenized in RIPA buffer containing protease and phosphatase inhibitors. The BCA assay was used to determine protein concentration. An equal amount of total proteins was separated by SDS-PAGE and transferred to PVDF membrane. After blocking, the membrane was then incubated with the following primary antibodies (rabbit anti-Fos polyclonal antibody (1 : 1000, TDY206, TDY biotech), rabbit anti-iNOS polyclonal antibody (1 : 500, ab15323, Abcam), mouse anti-GABAb (1 : 500, ab55051, Abcam), and mouse anti-*β*-actin (1 : 5000, TDY041, TDY biotech)) at 4°C overnight, respectively. After washing three times with PBS supplemented with 0.1% Tween, the membrane was incubated with appropriate secondary antibody (HRP-labeled goat anti-rabbit IgG (H + L) (1 : 20000, S004, TDY biotech), HRP-labeled goat anti-mouse IgG (H + L) (1 : 20000, S001, TDY biotech)) for 1 h at room temperature. The protein signals were developed with the SuperSignal West Dura Extended Duration substrate (Pierce) and imaged using the ChemiDoc^TM^ XRS + system (Bio-Rad).

### 2.10. Enzyme-Linked Immunosorbent Assay

Twenty minutes after the behavioral and electromyographic testing, rats were deeply anesthetized with sodium pentobarbital (100 mg/kg intraperitoneal). The T8-T10 dorsal spinal cord was removed. The dorsal spinal cord was grinded in liquid nitrogen with a mortar and pestle. The protein concentrations of cGMP and PKG proteins were quantified by ELISA according to the manufacturer's protocol. The cGMP ELISA kit (KGE003) was purchased from R&D systems, and the PKG ELISA kit (EY-(Ela)-2763) was obtained from Shanghai Institute of biotechnology Co., Ltd.

### 2.11. Statistical Analysis

The statistical analyses were performed using GraphPad Prism version 8 (GraphPad Software Inc., USA). The data of body weight, 3-h food intake, AWR score, and EMG activity were presented as mean ± SEM using two-way ANOVA analysis with repeated measures followed by Tukey's multiple comparisons test to compare the differences between two groups. The other data were presented as mean ± SEM using one-way ANOVA analysis followed by Tukey's multiple comparisons test to compare the differences between two groups. A *P* value <0.05 was considered to be statistically significant.

## 3. Results

### 3.1. SNS Did Not Improve the Weight Loss of FD Model Rats

**Figure 2:**
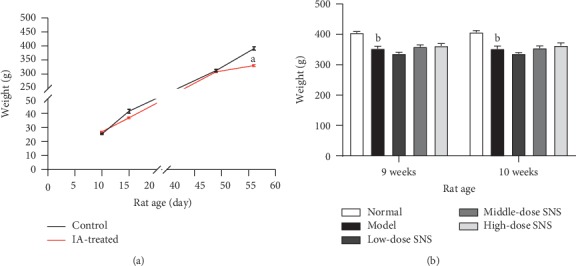
(a) Effects of IA treatment (10 days to 15 days) and tail-clamping (49 days to 56 days) on the weight of rats (*n* = 6 in the control group and *n* = 24 in the IA-treated group). (b) Effect of SNS treatment (9-week to 10-week) on the weight of rats (*n* = 6/group). Data are expressed as mean ± SE. ^*a*^*P* < 0.001 vs. control group; ^*b*^*P* < 0.001 vs. normal group.

The effects of IA-treatment, tail-clamping, and SNS treatment on rats' weight are shown in Figure 2. Before IA-treatment, there was no significant difference in the baseline weight between the control group and the IA-treated group. Although rats in model group lost some weight during IA treatment (10-day to 15-day), there was no statistical difference between IA-treated group and the control group at the age of 15 days as well as 7 weeks (49 days). However, after 7 days of tail-clamping (49-day to 56-day), the weight of rats in the IA-treated group was significantly lower than that in the control group (*P* < 0.001). At the age of 9 weeks and 10 weeks, there was no statistical difference among the low-dose SNS group, middle-dose SNS group, high-dose SNS group, and model group, which indicated that 7 days of SNS treatment was not effective in improving the weight loss of FD model rats.

### 3.2. Middle-Dose SNS Increased the 3-h Food Intake of FD Model Rats

**Figure 3:**
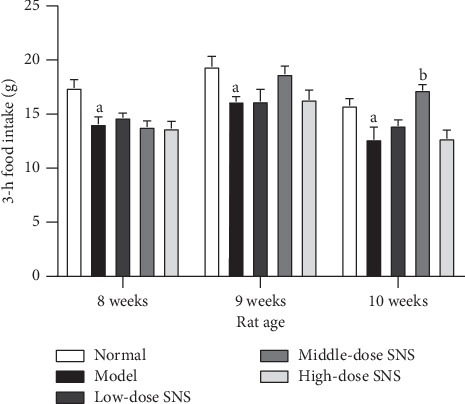
The 3-h food intake of rats at the age of 8 weeks, 9 weeks, and 10 weeks. Data are expressed as mean ± SE and *n* = 6/group. ^*a*^*P* < 0.05 vs. control group, ^*b*^*P* < 0.001 vs. model group.

At the age of 8 weeks, 9 weeks, and 10 weeks, the 3-h food intake in FD model rats was significantly lower than that in normal rats (*P* < 0.05). At the age of 10 weeks, the 3-h food intake in rats from the middle-dose SNS group was significantly higher than that in the model group (*P* < 0.001). However, high-dose SNS and low-dose SNS did not significantly improve the 3-h food intake in FD model rats (Figure 3).

### 3.3. SNS Slowed Down the Increasing Trend of AWR Score in Response to Graded Gastric Distention of FD Model Rats

With the increase of graded gastric distention, rats' AWR scores increased gradually. Each group's AWR scores in response to the gastric distension of 60 mmHg and 80 mmHg were significantly increased compared to its own AWR score in response to the distension of 20 mm Hg, respectively (*P* < 0.05 or *P* < 0.001). However, the increasing trend of AWR score in the model group was more obvious, which began to show statistically significant difference from the distension of 40 mmHg (*P* < 0.05) (Table 2).

### 3.4. SNS Decreased the EMG Activity in the Acromiotrapezius Muscle of FD Model Rats

**Figure 4:**
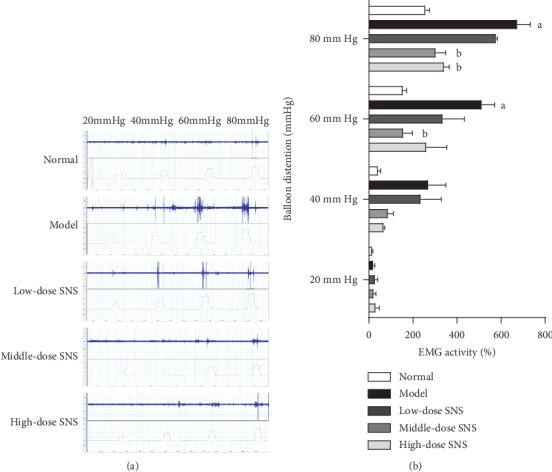
Effects of SNS on the EMG activity in rats. (a) Representative photographs of EMG records under graded gastric balloon distention. (b) Quantification of EMG activity (the rate of change of RMS value of the EMG signal). Data are expressed as mean ± SE and *n* = 6/group. ^*a*^*P* < 0.01 vs. normal group with the same graded distention; ^*b*^*P* < 0.01 vs. model group with the same graded distention.

The EMG activity was determined by the rate of change of RMS value of the EMG signal in the acromiotrapezius muscle in response to graded balloon distension (Figure 4). The EMG activity in the model group was significantly increased compared with the normal group at distention of 60 mm Hg (*P* < 0.01) and 80 mm Hg (*P* < 0.01). The EMG activity of the middle-dose SNS-treated group was significantly reduced compared to that of the model group at distention of 60 mm Hg (*P* < 0.01) and 80 mm Hg (*P* < 0.01). The EMG activity in the high-dose SNS-treated group was significantly reduced compared to the model group at a distention of 80 mm Hg (*P* < 0.01).

### 3.5. SNS Reduced the Intensity of Immunostaining of iNOS in the Spinal Dorsal Horn

**Figure 5:**
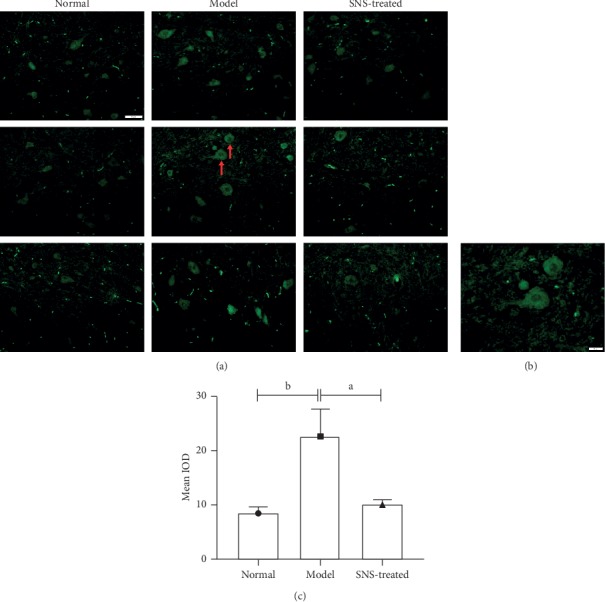
Spinal iNOS-positive cells were observed by immunofluorescence. (a) Representative images of spinal dorsal horn sections of immunofluorescent labeling iNOS after behavioral and electromyographic tests, scale bar = 50 *μ*m. (b) Higher magnification of representative positive cells in (a), scale bar = 20 *μ*m. (c) Quantification of iNOS immunoreactivity (value of mean IOD). Data were expressed as mean ± SE and *n* = 4/group. ^*a*^*P* < 0.05, ^*b*^*P* < 0.01.

To explore the possible mechanisms of SNS regulating the expression of iNOS in the spinal dorsal horn of FD model rats, the middle-dose group (optimal effective group) was used to further study as the SNS-treated group. Spinal cord sections were stained with antibodies specific to iNOS. As shown in Figure 5, the immunoreactivity was increased in FD model rats, and such induction was greatly reduced by SNS treatment.

### 3.6. SNS Downregulated the Expression of the c-Fos Gene in the Spinal Dorsal Horn of FD Model Rats

**Figure 6:**
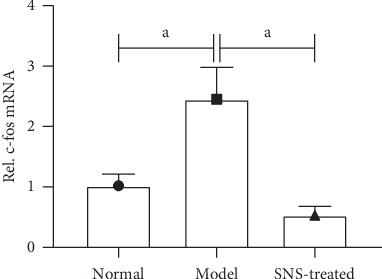
Effect of SNS on the expression of the c-fos gene in FD model rats. The Level of c-fos gene was determined using real-time quantitative PCR. Data are expressed as mean ± SE and *n* = 4/group. ^*a*^*P* < 0.001.

The expression of the c-fos gene in the T8-T10 spinal dorsal horn was determined using real-time quantitative PCR. As shown in Figure 6, IA-treatment and tail-clamping significantly increased the expression of the c-fos gene in the T8-T10 spinal dorsal horn of rats, whereas SNS treatment significantly downregulated the expression of the c-fos gene of FD model rats.

### 3.7. SNS Inhibited the NO-cGMP-PKG Signal in FD Model Rats

**Figure 7:**
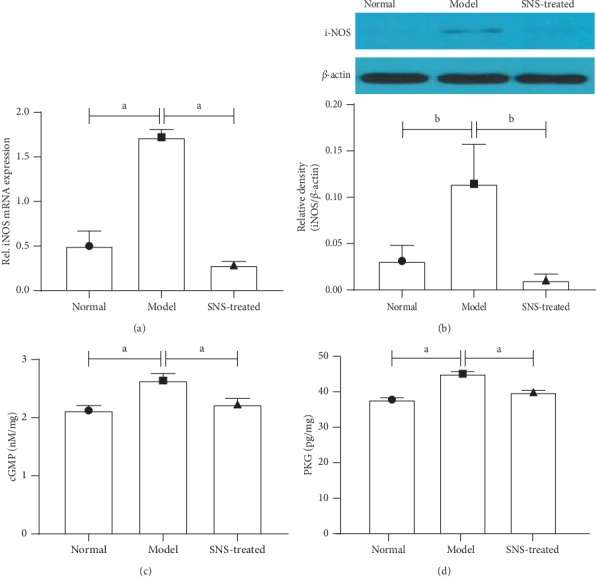
SNS inhibited the NO-cGMP-PKG signal in FD model rats. The expression of iNOS was tested using real-time quantitative PCR (a) and western blot (b) (*n* = 4/group). The levels of cGMP (c) and PKG (d) were measured by enzyme-linked immunosorbent assay (*n* = 6/group). Data are expressed as mean ± SE. ^*a*^*P* < 0.001, ^*b*^*P* < 0.01.

Real-time quantitative PCR and western blot were used to test the expression of iNOS. As shown in Figures 7(a) and 7(b), the expression of iNOS was significantly upregulated in the model group compared to that in the normal group, whereas SNS treatment significantly reduced the expression of iNOS in rats with FD. Similar with the change of iNOS, the levels of cGMP and PKG in T8-T10 spinal dorsal horn were significantly increased in the model group, and SNS treatment decreased these levels (Figures 7(c) and 7(d)).

### 3.8. SNS Restored the Expression of GABAb in the Spinal Dorsal Horn of FD Model Rats

**Figure 8:**
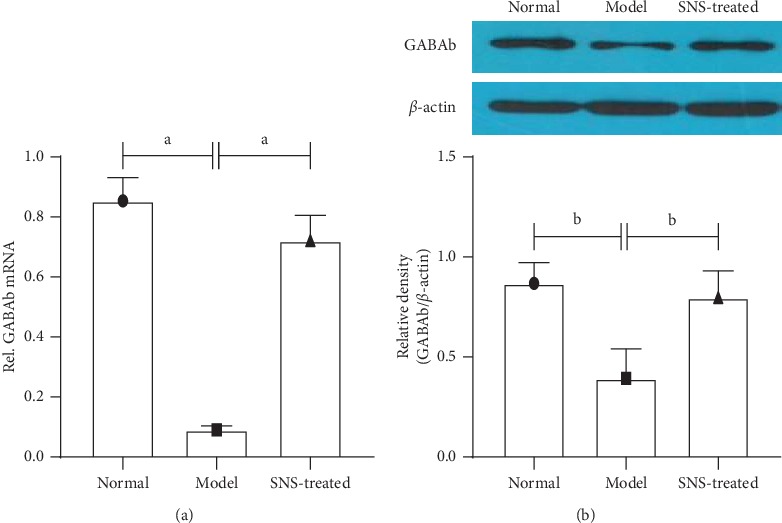
Expression of GABAb in mRNA level was tested using real-time quantitative PCR. (a) The expression of GABAb in protein level was tested using western blot. (b) Data are expressed as mean ± SE and *n* = 4/group. ^*a*^*P* < 0.001, ^*b*^*P* < 0.001.

The expression of GABAb in mRNA and protein levels were significantly downregulated in the spinal dorsal horn of FD model rats, and SNS treatment restored the expression of GABAb to normal level (Figure 8).

## 4. Discussion

Visceral hypersensitivity is an important pathological mechanism of FD, which contributes to the upper gastrointestinal symptoms, such as abdominal distention, postprandial fullness, early satiety, and upper abdominal pain [22]. Patients with FD tend to be hypersensitive to gastric distension and hyperalgesia [23]. According to TCM, “spleen-deficiency and qi-stagnation” is the most common syndrome of FD. SNS is commonly used to improve symptoms of abdominal distension and abdominal pain by invigorating spleen and regulating qi. In this study, SNS improved the 3-h food intake and alleviated the behavioral and visceromotor responses to gastric distention in FD model rats. Besides, it also reduced the iNOS expression and regulated the NO/cGMP/PKG signaling pathway in the spinal dorsal horn of rats.

Animal models have a critical role in drug screening and the development of therapies for human diseases. Accordingly, it is essential to establish an FD model compliant with the pathogenesis of TCM syndrome for the study of TCM treatment. Previous studies [21, 24] have shown that neonatal IA-treated rats exhibit a gastric hypersensitivity to adulthood, which is similar to the long-term formation process of “spleen-deficiency” in TCM. Recently, another FD model was established by a short-term tail-clamping approach, which can partly simulate the formation process of “qi-stagnation” [19]. In this study, IA-lavage was used to mimic the trigger factor of spleen-deficiency, and tail-clamping was used to mimic the trigger factor of qi-stagnation. As a result, the syndromes of “spleen-deficiency and qi-stagnation” were simulated.

Most FD patients who are hypersensitive to gastric distention experience symptoms of epigastric pain and weight loss [25, 26]. In this study, the body weight and 3-h food intake in FD model rats were significantly lower than those in normal rats, which was consistent with the behavioral scores and EMG activity results in rats. SNS significantly increased the 3-h food intake in FD model rats and alleviated the behavior and visceromotor responses to gastric distention. Yet, SNS did not improve the weight loss in FD model rats. Even though the visceral hypersensitivity caused by neonatal IA treatment persisted to adulthood, which was consistent with previous studies [21, 24], the weight loss caused by neonatal IA treatment was not statistically significant, especially when the rats grew up. This suggests that the relationship between weight loss and visceral hypersensitivity might not be inevitable although they both occurred in our FD model rats. It may also partly explain why SNS improved the visceral hypersensitivity but did not improve the weight loss in FD model rats.

To further confirm the hyperalgesia of FD rats to gastric distention, we detected the c-fos gene in the spinal dorsal horn of rats. As a product of the immediate early gene, the c-fos protein was regarded as a pain marker in the spinal cord [27]. Our results showed that the expression of the c-fos gene in the spinal dorsal horn was significantly upregulated in FD model rats compared to normal rats, and SNS treatment restored the changes. This indicated that the pain signal in FD model rats was stronger compared to that in normal rats, and SNS could regulate their hyperalgesia, which was consistent with our previous results on behavioral scores and EMG activity in rats.

In order to explore the intrinsic mechanism of SNS in the treatment of FD and visceral hypersensitivity, we performed additional analyses. Visceral hypersensitivity is a highly complex and subjective phenomenon associated with multiple levels of the nervous system and a wide range of neurotransmission. NO/cGMP/PKG signaling pathway is widely implicated in hyperalgesia [28]. Nitric oxide (NO) is predominantly produced along the biosynthetic process catalyzed by nitric oxide synthases (NOS), and iNOS is one of the nitric oxide synthases that is only expressed when induced by pathological stimuli. The primary action of NO is to activate the soluble form of the enzyme guanylate cyclase (GC), which has been designated as a physiological NO receptor. Functionally, soluble GC directly leads to the formation of cyclic guanosine monophosphate (cGMP) and in turn, to the activation of the cGMP-dependent protein kinase (PKG) [29] phosphorylation of some membrane proteins and increase of the excitability of these cells. This process elevates the response of these spinal dorsal horn cells to afferent impulses, thus inducing hyperalgesia [30]. Our results demonstrated that SNS might ameliorate the visceral hypersensitivity by inhibiting the expression of iNOS, decreasing the formation of NO, and inhibiting the activation of NO/cGMP/PKG signaling pathway in the dorsal horn of the spinal cord.

Besides, our results also showed that SNS treatment recovered the expression of GABAb in gene and protein levels in the dorsal horn of the spinal cord in FD model rats. The amino acid *γ*-aminobutyric acid (GABA) is a major inhibitory neurotransmitter in the central nervous system that functions through one of its receptor complexes, either the ionotropic GABAa or the metabotropic GABAb. Activation of GABAb results in significant analgesia, which possibly occurs through presynaptic inhibition of neurotransmitter release from the central endings of primary nociceptors in the spinal cord [24, 31]. Several studies have demonstrated that the GABAergic pathway is a potential target for the treatment of functional dyspepsia and related conditions [24, 32]. Furthermore, there is a significant link between the GABAergic pathway and NO pathway. On one hand, NO can inactivate GABAb receptor by phosphorylation through the NO-cGMP pathway, thus decreasing its inhibition [33]. On the other hand, GABA can inhibit NO neurons through GABA receptor and reduce the release of NO, thereby reducing the levels of cGMP [34]. In this study, SNS regulated the expression of GABAb receptor in the spinal dorsal horn, which might be another way through which SNS regulated the NO/cGMP/PKG pathway and ameliorated the visceral hypersensitivity of FD model rats.

## 5. Conclusions

Our results demonstrated that SNS alleviates the visceral hypersensitivity in FD model rats. Regulation of the NO/cGMP/PKG signaling pathway in the spinal dorsal horn may be the intrinsic mechanism generating SNS benefits.

## Figures and Tables

**Table 1:** Primers used in this study.

Primer	Sequence (5′-3′)
inos_PF	CAGCATCCACGCCAAGAACG
inos_PR	CACAGTTTGGTCTGGCGAAG
fos_PF	CCGAAGGGAAAGGAATAAGATG
fos_PR	GGGCTGCCAAAATAAACTCC
gabab_PF	TGGCACTGGCTGCTGTCTTCC
gabab_PR	CACTCCTTCTTCTCCTCCTTCTTCG
gapdh_PF	TGGAGTCTACTGGCGTCTT
gapdh_PF	TGTCATATTTCTCGTGGTTCA

**Table 2:** Abdominal withdrawal reflex score of rats (mean ± SE, *n* = 6/group).

Group	20 mm Hg	40 mm Hg	60 mm Hg	80 mm Hg
Normal	0.33 ± 0.21	1.00 ± 0.26	2.17 ± 0.31^*a*^	3.17 ± 0.31^*a*^
Model	0.33 ± 0.21	1.83 ± 0.31^*a*^	3.33 ± 0.33^*a*^	3.83 ± 0.17^*b*^
Low-dose SNS	0.50 ± 0.22	1.17 ± 0.17	3.33 ± 0.21^*b*^	3.82 ± 0.17^*b*^
Middle-dose SNS	0.33 ± 0.21	1.00 ± 0.26	3.00 ± 0.26^*b*^	3.17 ± 0.31^*a*^
High-dose SNS	0.33 ± 0.21	1.00 ± 0.26	3.00 ± 0.26^*a*^	3.50 ± 0.22^*b*^

^*a*^ *P* < 0.01, ^*b*^*P* < 0.001, vs. the distension of 20 mm Hg in the same group.

## Data Availability

All data included in this study are available from the corresponding author upon request.
